# 한국 응급실 간호사의 임종간호 태도에 미치는 영향요인: 횡단적 조사 연구

**DOI:** 10.7586/jkbns.24.032

**Published:** 2024-11-21

**Authors:** Jeong-Eun Park, Hye-Jin Kim

**Affiliations:** 1 Ulsan University Hospital, Ulsan, Korea; 1 울산대학교 병원; 2 Department of Nursing, University of Ulsan, Ulsan, Korea; 2 울산대학교 간호학과

**Keywords:** Attitude to death, Emergency nursing, Empathy, Perception, Terminal care, 죽음에 대한 태도, 응급 간호, 공감, 인식, 임종간호

## Abstract

**Purpose:**

This study explored the relationships of emergency room nurses’ perceptions of a good death, sense of calling, empathy competence, and end-of-life care stress with attitudes toward end-of-life care. It also identified the factors influencing attitudes toward end-of-life care.

**Methods:**

Data collection was conducted from September 1 to September 27, 2023 among emergency room nurses from general hospitals in Korea. A total of 248 nurses were included in the final analysis. A structured questionnaire was used to collect data. Data analysis was performed using the SPSS Win 26.0 program.

**Results:**

The study found positive correlations between attitudes toward end-of-life care and perceptions of a good death (r = .48, *p* < .001), sense of calling (r = .33, *p* < .001), and empathy competence (r = .24, *p* < .001). Conversely, a negative correlation was observed between end-of-life care stress and attitudes toward end-of-life care (r = -.44, *p* < .001). Multiple regression analysis revealed that an experience of a family member`s death, perceptions of a good death, a sense of calling, and end-of-life care stress were factors that significantly influenced attitudes toward end-of-life care among the emergency room nurses.

**Conclusion:**

In order to foster positive end-of-life care attitudes among emergency room nurses, it is essential to provide education on perceptions of a good death. Systematic education can effectively alleviate end-of-life care stress and help nurses better support this important aspect of practice.

## 서론

### 1. 연구의 필요성

2023년 한국 전체 인구의 18.2%가 65세 이상 고령인구로, 2025년에는 한국이 OECD 국가 중 가장 빠른 속도로 초고령사회로 진입할 것으로 전망된다[1]. 이러한 가운데 2023년도 보건복지부 노인실태조사[2]에 의하면 65세 이상 노인 중 86.1%가 만성질환을 가지고 있고, 3가지 이상 만성질환을 가진 복합만성질환군이 35.9%에 달하고 있어 만성질환 합병증으로 인한 응급실 내원 빈도가 높아지고 있다. 또한 응급의료 통계연보[3]에 따르면 2022년 응급실에 방문한 노인환자 중 60대 사망률은 17.5%, 70대 24.3%, 80대 이상 40.6%로 응급실 방문환자의 사망률이 고령일수록 높아지고 있어, 응급실에서 임종간호 수행이 필수적임을 알 수 있다.

죽음에 직면한 환자와 가족에게 좀 더 의미있는 임종간호를 제공하기 위해서는 간호사의 임종간호 태도 또한 긍정적일 필요가 있는데, 긍정적인 임종간호 태도를 가진 간호사는 환자에게 전인적인 임종간호를 수행함으로써 환자가 좋은 죽음을 맞이할 수 있도록 도와준다[4].

임종간호를 제공하는 간호사가 환자의 생애 말기 신체적, 정서적, 사회적 측면을 모두 충족시키는 좋은 죽음[5]을 인식한다면, 임종을 앞둔 환자가 존엄하고 편안한 죽음을 맞이할 수 있도록 도울 수 있다[4,6,7]. 더불어 간호사는 임종간호를 제공하면서 임종환자 돌봄의 의미를 깨닫게 되고, 임종간호가 전문직에서 수행할 수 있는 고유한 역할이라는 자부심과 소명의식을 가지게 된다[8]. 소명의식은 자신의 일에 대한 목표를 추구하며 사회의 공공선에 긍정적인 영향을 미치려는 태도로[9], 소명의식이 있는 간호사는 책임감을 가지고 임종환자에게 질 높은 임종간호를 제공할 수 있다[10]. 이에 소명의식이 임종간호태도에 영향을 미칠 수 있음을 고려해야 한다. 간호사의 공감역량 또한 임종간호 태도에 영향을 줄 수 있는 요인 중 하나이다. 간호사는 공감역량을 통해 죽음을 앞둔 환자와 그 가족이 평온한 마지막 순간을 맞이할 수 있도록 총체적 돌봄을 제공할 수 있다[11]. 이에 임종환자 곁에 있는 간호사는 환자와 보호자를 응대하면서 공감능력이 있고, 숙련되고, 지식이 풍부하고 문화적으로 유능해야 한다[12].

간호사의 임종간호 스트레스도 임종간호 태도에 유의한 변수로 작용하기 때문에[13] 응급실 간호사의 임종간호 스트레스 정도를 확인하고, 임종간호 태도에 미치는 영향을 확인하는 것이 중요하다. 임종간호 스트레스란 간호사가 겪는 임종환자에 대한 심리적 고통, 신체적 무력감을 말하며[14], 많은 업무수행을 하면서 동시에 한 사람의 임종을 존엄하게 맞이할 수 있도록 도와야 한다는 점에서 일반 간호업무 스트레스와는 다르다[15]. 더욱이 응급실 간호사는 긴박한 환경에서 소생간호와 임종간호를 동시에 제공해야 하므로 응급실 간호사의 임종간호 스트레스는 타부서와 다를 것으로 예상된다.

간호사를 대상으로 한 국내 선행연구에서 좋은 죽음에 대한 인식[12], 공감역량[11], 임종간호 스트레스[15]와 간호사의 임종간호 태도에 대한 선행연구[11,12,15]는 있었으나, 간호사의 필수 소양인 소명의식과 임종간호 태도와 직접 관련된 연구는 찾아볼 수 없었다. 또한 응급실 간호사의 임종간호 태도 관련 연구를 찾기 어려웠다. 이에 본 연구에서는 응급실 간호사의 임종간호 태도에 영향을 미치는 요인을 확인하기 위해, 좋은 죽음에 대한 인식, 소명의식, 공감역량, 임종간호 스트레스와 임종간호 태도를 파악하고자 한다. 이를 통해 응급실 간호사들의 긍정적인 임종간호 태도를 촉진하기 위한 중재 개발의 기초자료를 제공하고자 한다.

### 2. 연구 목적

본 연구는 응급실 간호사의 좋은 죽음에 대한 인식, 소명의식, 공감역량, 임종간호 스트레스, 임종간호 태도를 파악하고, 임종간호 태도에 영향을 미치는 요인을 확인하고자 한다. 구체적인 목적은 다음과 같다.

1) 응급실 간호사의 좋은 죽음에 대한 인식, 소명의식, 공감역량, 임종간호 스트레스, 임종간호 태도를 파악한다.

2) 응급실 간호사의 좋은 죽음에 대한 인식, 소명의식, 공감역량, 임종간호 스트레스, 임종간호 태도 간의 상관관계를 분석한다.

3) 응급실 간호사의 좋은 죽음에 대한 인식, 소명의식, 공감역량, 임종간호 스트레스가 임종간호 태도에 미치는 영향요인을 확인한다.

## 연구 방법

### 1. 연구 설계

본 연구는 응급실 간호사의 좋은 죽음에 대한 인식, 소명의식, 공감역량, 임종간호 스트레스, 임종간호 태도를 파악하고, 임종간호 태도에 영향을 미치는 요인을 확인하기 위한 서술적 조사연구이다.

### 2. 연구 대상

본 연구는 경상권 소재 8개 종합병원(500병상 이상) 응급실에 종사하는 응급실 경력 1년 이상의 일반 간호사 중 응급실에서 임종간호를 1회 이상 수행한 간호사를 대상으로 하였다. 독자적인 임종간호 수행에 어려움이 있는 1년 미만의 신규 간호사와 직접적으로 환자간호를 수행하지 않는 수간호사 이상 관리자는 제외하였다[13].

적정 표본 수 산출을 위해 G*power 3.1.2 program을 이용하였다. 선행연구[13]에 근거한 중간효과크기 .15, 유의수준 .05, 검정력 .95, 일반적 특성 및 연구변수 19개를 적용하여 다중회귀분석에 필요한 최소 표본 수는 217명이 산출되었다. 이에 탈락률 약 20%를 고려하여 총 269명의 간호사에게 설문지를 배부하였고, 250부를 회수하였으며, 이 중 응답이 미비한 2부를 제외한 총 248부의 설문지가 최종분석되었다.

### 3. 연구 도구

#### 1) 일반적 특성

일반적 특성은 연령, 결혼상태, 학력, 종교, 가족 죽음 경험 여부, 총 임상경력, 응급실 근무경력, 임종간호 경험 횟수, 임종간호 시 환자와 보호자에게 임종에 대한 설명 및 교육을 제공하기 위한 노력 여부, 임종간호에 대한 교육 이수 경험 여부 총 10항목을 조사하였다.

#### 2) 좋은 죽음에 대한 인식

좋은 죽음에 대한 인식은 Schwartz 등[16]이 개발한 The concept of a good death measure를 원저자로부터 한국어판을 구입하여 사용하였다. 본 도구는 친밀감, 통제감, 임상증상 3개의 하위영역으로, 총 17문항으로 구성되었다. Likert 4점 척도로 점수가 높을수록 간호사의 좋은 죽음 인식이 높음을 의미한다. Schwartz 등[16] 연구의 신뢰도는 .87, 본 연구에서는 .87이었다.

#### 3) 소명의식

소명의식은 Dik 등[9]이 개발한 The Calling and Vocation Questionnaire (CVQ)를 Shim과 Yoo [17]가 번안, 타당화한 한국어판 CVQ-K로 저자들의 승인을 얻어 사용하였다. 본 도구는 초월적인 부름, 목적과 의미, 친사회적 지향 3개의 하위영역으로 이루어져 있으며, 총 12문항으로 구성되었다. Likert 5점 척도로 점수가 높을수록 간호사의 소명의식이 높음을 의미한다. Shim과 Yoo [17] 연구의 신뢰도는 .85이었고, 본 연구의 신뢰도는 .89이었다.

#### 4) 공감역량

공감역량은 Lee [18]가 개발한 간호사의 공감역량 측정도구를 저자의 승인을 얻어 사용하였다. 본 도구는 소통력, 민감성, 통찰력 3개의 하위영역으로 이루어져 있으며, 총 17문항으로 구성되었다. Likert 5점 척도로 점수가 높을수록 간호사의 공감역량이 높음을 의미한다. Lee [18]연구의 신뢰도는 .93이었고, 본 연구의 신뢰도는 .90이었다.

#### 5) 임종간호 스트레스

임종간호 스트레스는 Lee [19]가 개발한 임종간호에 대한 스트레스 측정 도구를 저자의 승인을 얻어 사용하였다. 본 도구는 전문지식과 기술 부족, 의료한계에 대한 갈등, 업무량 과중, 임종환자에 대한 시간 할애의 어려움, 임종 환자 간호에 대한 부담감, 임종 환자의 인간적 갈등, 환자 및 보호자의 임종에 대한 부정적 태도 7개의 하위영역으로 이루어져 있으며, 총 40문항으로 구성되었다. Likert 5점 척도로 점수가 높을수록 간호사의 임종간호 스트레스 정도가 높다는 것을 의미한다. Lee [19]의 신뢰도는 .93이었고, 본 연구에서의 신뢰도는 .97이었다.

#### 6) 임종간호 태도

임종간호 태도는 Frommelt [20]가 개발한 Frommelt Attitudes toward Care of the Dying Scale을 Cho와 Kim [21]이 번역하여 수정한 도구로, 저자들의 승인을 얻어 사용하였다. 총 30문항으로, Likert 4점 척도로 점수가 높을수록 간호사의 임종간호 태도가 긍정적인 것을 의미한다. Cho와 Kim [21]의 신뢰도는 .86이었고, 본 연구에서는 .92이었다.

### 4. 자료 수집 및 분석

2023년 9월 1일부터 9월 27일까지 경상권 소재 500병상 이상 종합병원 8곳(울산대학교병원, 고신대학교복음병원, 인제대학교 부산백병원, 인제대학교 해운대백병원, 영남대학교병원, 포항세명기독병원, 부산대학교병원, 동강병원)을 연구자가 방문하여 자료 수집하였다. 간호부에 연구의 목적과 내용에 대해 설명하고, 연구 허가를 받은 후 간호부를 통해 설문지를 배부하고 회수하였다. 회수된 250부 중 응답이 미비한 2부를 제외하고, 248부가 분석에 사용되었다.

수집된 자료는 SPSS Win 26.0 (IBM Corp., Armonk, NY, USA ) 프로그램을 이용하여 분석하였다. 대상자의 일반적 특성에 따른 좋은 죽음에 대한 인식, 소명의식, 공감역량, 임종간호 스트레스, 임종간호 태도의 차이는 독립표본 t-검정과 분산분석을 실시하였고, 사후검정으로 Scheffé test를 실시하였다. 대상자의 좋은 죽음에 대한 인식, 소명의식, 공감역량, 임종간호 스트레스, 임종간호 태도의 관계는 피어슨 상관계수 값으로 분석하였다. 응급실 간호사의 임종간호 태도에 영향력이 있는 변수를 파악하고자 다중회귀분석을 이용하였다. 단변량 분석에서 임종간호태도와 유의한 관련성이 있는 것으로 나타난 가족 죽음 경험, 임종에 대한 설명 및 교육 노력, 임종간호 경험 횟수, 그리고 좋은 죽음에 대한 인식, 소명의식, 공감역량, 임종간호 스트레스를 예측변수로 하여 다중회귀분석을 실시하였다. 예측변수 중 가족 죽음 경험은 가변수(dummy variable) 처리하였고, 임종에 대한 설명 및 교육 노력과 임종간호 경험 횟수는 연속변수로 투입하였다.

### 5. 윤리적 고려

본 연구는 연구기관의 임상연구심의위원회 승인(IRB No. UUH 2023-07-005-002)을 받은 후 진행하였다. 연구동의서를 읽고 자발적으로 연구에 참여하기로 한 대상자에 한하여 설문조사를 진행했으며, 연구동의서에는 연구의 목적, 절차 및 익명성 보장, 연구 참여 중단 시 불이익 없음 등에 대한 내용을 기술하였다. 설문지 응답이 완료된 후 대상자에게 소정의 답례품을 제공하였다. 응답이 완료된 설문지는 대상자의 자율성과 익명성 보장을 위해 밀봉된 봉투에 보관되었으며, 연구자가 직접 간호부에 방문하여 수거하였다. 수집된 자료는 개인정보보호법에 따라 관리할 것이고, 연구 종료 후 3년간 보관 이후 문서를 파쇄하여 영구적으로 삭제할 예정이다.

## 연구 결과

### 1. 대상자의 일반적 특성에 따른 좋은 죽음에 대한 인식, 소명의식, 공감역량, 임종간호 스트레스 및 임종간호 태도

대상자 중 25∼29세가 140명(56.5%)으로 가장 많았고, 미혼이 193명(77.8%), 학사 이상인 경우가 191명(77.0%), 무교인 경우가 184명(74.2%)으로 가장 많았다. 총 임상경력은 1∼4년이 124명(50.0%)으로 가장 많았으며, 최근 3개월간 임종간호 경험 횟수는 1∼4회가 127명(51.2%)으로 가장 많았다. 임종간호 시 환자, 보호자에게 간호사가 임종에 대한 설명 및 교육 노력 정도는 ‘노력하는 편이다’라고 응답한 대상자가 187명(75.4%)으로 가장 많았다(Table 1).

대상자의 좋은 죽음에 대한 인식은 4점 만점에 평균 2.99 ± 0.43점, 소명의식은 5점 만점에 평균 3.12 ± 0.65점, 공감역량은 5점 만점에 평균 3.63 ± 0.49점이었다. 임종간호 스트레스는 5점 만점에 평균 3.27 ± 0.84점이었으며, 임종간호 태도는 4점 만점에 평균 2.87 ± 0.51점이었다(Table 2).

대상자의 일반적 특성에 따른 좋은 죽음에 대한 인식은 유의한 차이가 없었다. 소명의식은 석사 이상인 경우 전문학사, 학사보다 높았다(*p* = .015). 종교가 있는 사람이 없는 사람보다(*p* = .018), 임종간호 시 환자, 보호자에게 임종에 대한 설명 및 교육을 하려고 매우 노력하는 사람이 노력하지 않는 사람보다 소명의식이 높았다(*p* = .004). 공감역량은 석사 이상인 경우 전문학사, 학사보다(*p* = .042), 임종간호 시 환자, 보호자에게 임종에 대한 설명 및 교육을 하려고 매우 노력하는 사람이 노력하지 않는 사람보다 높았다(*p* < .001). 임종간호 스트레스는 40세 이상이 25∼29세보다(*p* = .042), 기혼이 미혼보다 높았다(*p* = .016). 총 임상경력이 15년 이상인 경우 1∼4년보다(*p* = .016), 임종간호 경험 횟수가 1∼4회인 경우가 15회 이상보다 높았다(*p* = .030). 임종간호 태도는 가족의 죽음을 경험한 경우, 경험이 없는 사람보다(*p* = .036), 임종간호 경험 횟수가 15회 이상인 경우 1∼4회인 경우보다 긍정적이었다(*p* = .012). 임종간호 시 환자, 보호자에게 임종에 대한 설명 및 교육을 하려고 매우 노력하는 사람이 전혀 노력하지 않는 사람보다 임종간호 태도가 긍정적이었다(*p* = .009) (Table 2).

### 2. 대상자의 좋은 죽음에 대한 인식, 소명의식, 공감역량, 임종간호 스트레스 및 임종간호 태도 간의 상관관계

대상자의 좋은 죽음에 대한 인식은 소명의식(r = .32, *p* < .001), 공감역량(r = .33, *p* < .001), 임종간호 태도(r = .48, *p* < .001)와 양의 상관관계가 있었다. 대상자의 소명의식은 공감역량(r = .32, *p* < .001), 임종간호 태도(r = .33, *p* < .001)와 양의 상관관계가 있었다. 대상자의 공감역량은 임종간호 태도(r = .24, *p* < .001)와 양의 상관관계가 있었다. 그러나 임종간호 스트레스와 좋은 죽음에 대한 인식(r = −.43, *p* < .001), 소명의식(r = −.14, *p* = .019), 공감역량(r = −.19, *p* = .003), 임종간호 태도(r = −.44, *p* < .001)는 음의 상관관계가 있었다(Table 3).

### 3. 대상자의 임종간호 태도에 영향을 미치는 요인

다중회귀분석을 위한 가정검증 결과, 임종간호 태도에 관한 전체 회귀모형의 적합성은 충족되었고(F = 19.72, *p* < .001), 각 변수들의 공차는 .70∼.97, 분산팽창요인 값도 1.03∼1.44로 10 미만으로 나타나 다중공선성 문제는 없는 것으로 확인되었다. 잔차 분석결과 표준화된 잔차의 범위는 –2.51∼2.01로 2에 가까워 등분산성을 만족하였고, 정규성도 확인하였다.

다중회귀분석 결과, 대상자의 임종간호 태도에는 가족의 죽음 경험(β = .11, *p* = .031), 좋은 죽음에 대한 인식(β = .27, *p* < .001), 소명의식(β = .18, *p* < .001), 임종간호 스트레스(β = −.27, *p* < .001)가 영향을 미치는 변수들로 확인되었으며, 임종간호 태도의 35.0% 설명력을 나타냈다(Table 4).

## 논의

본 연구는 응급실 간호사의 좋은 죽음에 대한 인식, 소명의식, 공감역량, 임종간호 스트레스, 임종간호 태도를 파악하고, 각 변수들 간의 관계를 분석하며, 임종간호 태도에 미치는 영향요인을 확인하고자 수행되었다. 본 연구 결과를 토대로 하여 다음과 같이 논의하고자 한다.

본 연구에서 응급실 간호사의 좋은 죽음에 대한 인식은 4점 만점에 평균 2.99점이었다. 이는 응급실 간호사를 대상으로 같은 도구를 사용한 연구[22] 2.95점, 중환자실 간호사를 대상으로 한 연구[23]의 3.05점과 비슷한 수준이었다. 좋은 죽음에 대한 인식의 하위영역별 평균 점수는 친밀감, 임상증상, 통제감 순으로 높게 나타나 선행연구 결과[22,23]와 일치하였다. 이를 통해 간호사는 임종간호 제공에 있어 환자 및 보호자와의 친밀한 유대관계를 중요하게 생각하고 있음을 알 수 있었으며, 좋은 죽음에 대한 인식의 통제감 영역 향상을 위해 간호사는 환자가 임종에 대한 의사결정에 직접 참여하고 존중받을 수 있도록 노력해야함을 알 수 있었다.

본 연구에서 응급실 간호사의 임종간호 태도는 4점 만점에 2.87점으로 상급종합병원 간호사를 대상으로 같은 도구를 사용한 연구[7]에서 2.90점, 종합병원 간호사를 대상으로 한 연구[13]에서 3.01점과 유사하다. 임종간호 태도를 묻는 문항에서 ‘간호사는 필요 시 임종환자가 면회나 면담을 할 수 있도록 허용해야 한다.’ 는 문항의 점수가 가장 높았고, ‘간호는 임종환자의 가족까지도 포함해야 한다.’, ‘가족들은 임종환자가 남은 삶을 의미 있게 마무리하도록 도와야 한다.’ 순으로 점수가 높았다. 이는 응급실 간호사들이 임종간호에서 가족의 중요성과 조용한 환경의 필요성을 이미 인식하고 있음을 나타내며, 임종간호 시 가족에게도 돌봄을 제공해야 한다고 생각하는 임종간호 태도를 반영하는 것이다. 그러나 한국 응급실에서 임종환자는 보통 소생실에 배정되는데, 소생실은 소생에 초점을 맞추어 설계되어 있어 프라이버시가 존중되지 않으며[24], 임종간호 과정에 가족이 참여하는데 무리가 있다. 따라서 소음이 가득한 응급실 환경을 개선하여[25], 임종환자와 가족들이 좋은 죽음을 맞이할 수 있도록 하는 것이 필요하다.

본 연구에서 일반적 특성에 따른 응급실 간호사의 임종간호 태도는 가족의 죽음을 경험한 경우, 임종간호 경험 횟수가 많은 경우, 임종간호 시 환자, 보호자에게 임종에 대한 설명 및 교육을 매우 하려는 경우에 그렇지 않은 경우보다 긍정적이었다. 또한 응급실 간호사의 임종간호 태도에 영향을 미치는 요인은 가족 죽음 경험, 좋은 죽음에 대한 인식, 소명의식, 임종간호 스트레스로 나타났다. 응급실 간호사의 가족 죽음 경험은 임종간호 태도의 영향요인으로, 가족의 죽음을 경험하거나, 임종간호 경험 횟수가 많은 경우 임종간호 태도가 긍정적이었다는 본 연구 결과는 최근 1년간 가족이나 지인의 죽음을 경험한 경우, 임종간호를 경험한 횟수가 많을수록 임종간호 태도가 긍정적이었다는 선행연구결과[26]와 일치하였다. 이같은 경험은 죽음에 대한 관여도가 높아지면서, 임종환자에 대한 이해도 높아지고[26], 반복되는 임종간호를 통해 임종간호 실무능력이 향상되어[7] 긍정적 임종간호 태도를 갖는데 도움이 된 것으로 사료된다.

응급실 간호사의 좋은 죽음에 대한 인식은 임종간호 태도에 강하게 영향을 미치는 요인으로, 좋은 죽음에 대한 인식이 높을수록 임종간호 태도가 긍정적이었다. 이는 간호사의 죽음인식이 임종간호 태도에 강력한 영향요인이라고 보고한 연구[4], 죽음의 긍정적 의미에 대한 인식이 높을수록 임종간호 태도가 높다고 보고한 연구[13]결과와 유사하다. 이는 좋은 죽음에 대한 인식을 지닌 간호사의 경우 환자의 임종과정을 지켜보면서 스스로 죽음에 대해 생각하고 삶의 의미와 가치를 깨닫게 되면서 성숙해지고[27], 환자가 존엄하고 편안한 죽음을 맞이할 수 있도록 도울 수 있기 때문이다[7]. 반면에 부정적인 죽음인식을 가진 간호사는 임종간호 시 부담감, 공포, 좌절감, 우울감과 스트레스를 경험하게 되고[26], 죽음을 회피하는 경향이 있어 임종환자 간호에 장애 요인이 될 수 있다[28]. 따라서 간호사들이 좋은 죽음에 대한 인식을 가질 수 있도록 하는 것이 중요한데, 특히 임종간호 경험이 없는 신규를 대상으로 좋은 죽음을 경험한 사람들의 이야기를 공유하고, 좋은 죽음에 대한 인식을 증진시키기 위한 교육이 필요하다.

응급실 간호사의 소명의식 또한 임종간호 태도의 영향요인으로, 소명의식이 높을수록 임종간호 태도가 긍정적이었는데, 소명의식이 임상수행능력과 양의 상관관계가 있다고 보고한 선행연구[29]와 맥락을 같이한다. 소명의식은 직업을 삶의 일부로 받아들이고, 성공적인 업무수행으로 자아실현을 통해 타인 지향적 가치와 삶의 목적을 달성하는 태도로[9], 임종간호 태도에도 긍정적인 영향을 미치는 것으로 사료된다. 소명의식은 내면적인 성향이 강하므로, 대학은 올바른 간호전문직관을 가질 수 있는 교육을 간호학생들에게 제공하여 인간의 삶에 대한 철학적 고찰을 하도록 해야한다[30]. 또한 병원 조직 차원에서는 간호사들이 직업을 통한 삶의 의미를 더욱 긍정적으로 인식할 수 있도록 직업에 대한 소명의식과 자긍심을 고취시킬 수 있는 활동들을 제공하는 것이 필요하다.

마지막으로 응급실 간호사의 임종간호 스트레스 또한 임종간호 태도의 강한 영향요인으로 확인되었으며, 임종간호 스트레스가 낮을수록 임종간호 태도가 긍정적이었는데, 이는 임종간호 태도가 좋을수록 임종간호 스트레스가 낮아진다는 선행연구[31]와 유사한 결과이다. 임종간호 시 간호사가 겪는 스트레스는 환자, 보호자와의 의사소통에 장애를 주게 되고[31], 부정적인 임종간호 태도를 야기하여 임종간호 수행에까지 영향을 줄 수 있다. 이에 임종간호 스트레스를 낮추기 위해 간호사 스스로 영적건강을 위한 자기 관리가 필요하며, 병원 내 임종간호팀을 구성하여 정보공유 및 협력을 통해 임종간호 시 수반되는 업무량 과중을 줄이고, 효율적인 인력 배치 및 환경 개선이 필요할 것으로 사료된다.

본 연구는 임종간호를 수행하는 응급실 간호사의 임종간호 태도를 높이기 위한 기초자료로서의 활용 및 후속연구의 근거를 제시하였다는데 의의가 있다. 지금까지 선행연구들을 보면 임종간호 태도에 대한 서술적 연구가 많았는데, 추후에는 응급실 간호사의 임종간호 태도를 높이기 위해 간호사의 좋은 죽음에 대한 인식과 소명의식을 높이고, 임종간호 스트레스를 감소시킬 수 있는 체계적인 프로그램 개발과 교육이 필요하겠다.

본 연구의 제한점은 다음과 같다. 먼저 임종간호 스트레스와 임종간호 태도를 측정하는 도구를 선별하는 과정에서 많은 도구를 검토하였지만, 응급실 간호사를 대상으로 측정하기 적절한 도구는 찾는데 제한점이 있었다. 이에 도구들의 일부 문항은 일반 병동 간호사들과 업무의 차이가 있는 응급실 간호사에게 적용하기에는 한계가 있다. 따라서 응급실 간호사의 근무 환경과 업무를 반영한 응급실 간호사의 임종간호 태도를 측정할 수 있는 도구개발을 제안한다. 또한 본 연구에서는 대상자의 성별이 조사되지 않아 성별에 따른 임종간호 태도의 차이를 확인하지 못한 아쉬움이 있다. 더불어 대상자의 대다수가 20대로 가족이나 지인의 죽음을 경험하지 못한 경우가 많아 죽음을 바라보는 관점과 가치관이 다를 수 있다. 그러므로 본 연구를 응급실 간호사를 대상으로 일반화하는데 어려움이 있다. 마지막으로 본 연구는 경상권 소재 종합병원 응급실 간호사를 대상으로 하였으며, 병원마다 환자의 중증도가 다르므로 연구결과를 일반화하여 확대 해석하기에는 무리가 있다. 이에 대상자 분포를 균일하게 하여 반복 연구를 제언한다.

## 결론

본 연구에서 응급실 간호사의 임종간호 태도에 영향을 주는 요인을 분석한 결과 응급실 간호사의 가족 죽음 경험, 좋은 죽음에 대한 인식, 소명의식, 임종간호 스트레스가 영향을 미치는 변수들로 확인되었다. 이에 임종간호 경험이 없는 간호대학생과 응급실 신규간호사를 대상으로 좋은 죽음에 대한 인식 교육과 임종간호 교육이 필요하며, 소명의식을 높이기 위하여 올바른 간호전문직관을 가질 수 있도록 기관의 조직적 차원의 노력이 필요하다. 응급실 간호사의 업무에 있어서 신체적 돌봄뿐만 아니라 정서적, 영적 돌봄을 포함한 전인적 임종간호에 대한 중요성을 강조할 필요가 있다. 또한 응급실 특성을 고려하여 응급실 간호사의 임종간호 태도를 높이기 위한 임종간호중재 지침 개발의 필요성을 제언한다.

## Figures and Tables

**Table 1.** General Characteristics of Participants (N = 248)

Characteristics	Categories	n (%)
Age (yr)	25~29	140 (56.5)
	30~34	75 (30.2)
	35~39	15 (6.0)
	≧ 40	18 (7.3)
Marital status	Unmarried	193 (77.8)
	Married	55 (22.2)
Education	Associate’s degree	47 (19.0)
	Bachelor’s degree	191 (77.0)
	Master’s degree	10 (4.0)
Religion	Yes	64 (25.8)
	No	184 (74.2)
Experience of a family member’s death	Yes	45 (18.1)
	No	203 (81.9)
Total clinical career (yr)	1~4	124 (50.0)
	5~9	71 (28.6)
	10~14	36 (14.5)
	≧ 15	17 (6.9)
Experience of emergency department work (yr)	1~4	150 (60.5)
	5~9	68 (27.4)
	10~14	25 (10.1)
	≧ 15	5 (2.0)
Number of end-of-life care experiences within 3 months	1~4	127 (51.2)
	5~9	53 (21.4)
	10~14	42 (16.9)
	≧ 15	26 (10.5)
Efforts to provide explanations and education about end-of-life care	Very hardworking	26 (10.5)
	Somewhat hardworking	187 (75.4)
	Somewhat un-hardworking	32 (12.9)
	Not hardworking at all	3 (1.2)
Experience of education about end-of-life care	Yes	84 (33.9)
	No	164 (66.1)

**Table 2.** Perceptions of a Good Death, Sense of Calling, Empathy Competence, End-of-Life Care Stress, and Attitudes toward End-of-Life Care according to General Characteristics (N = 248)

Characteristics	Categories	Perceptions of a good death		Sense of calling		Empathy competence		End-of-life care stress		Attitudes toward end-of-life care	
		M ± SD, t/F(*p*)									
Age (yr)	25~29^a^	3.00 ± 0.41	0.58 (.627)	3.13 ± 0.68	0.93 (.426)	3.62 ± 0.51	0.87 (.457)	3.18 ± 0.82	2.77 (.042) d > a	2.89 ± 0.52	2.24 (.084)
	30~34^b^	3.01 ± 0.47		3.07 ± 0.61		3.58 ± 0.47		3.32 ± 0.89		2.92 ± 0.48	
	35~39^c^	2.93 ± 0.43		3.10 ± 0.65		3.67 ± 0.43		3.34 ± 0.82		2.68 ± 0.57	
	≧ 40^d^	2.88 ± 0.33		3.35 ± 0.65		3.78 ± 0.53		3.76 ± 0.65		2.65 ± 0.53	
Marital status	Unmarried	3.02 ± 0.43	1.88 (.061)	3.13 ± 0.67	0.46 (.646)	3.63 ± 0.49	0.12 (.898)	3.20 ± 0.85	−2.43 (.016)	2.89 ± 0.52	1.06 (.290)
	Married	2.89 ± 0.39		3.09 ± 0.60		3.62 ± 0.50		3.51 ± 0.77		2.81 ± 0.50	
Education	Associate’s degree^a^	3.00 ± 0.37	1.21 (.299)	3.05 ± 0.67	4.25 (.015) a,b < c	3.63 ± 0.47	3.20 (.042) a,b < c	3.20 ± 0.77	1.41 (.245)	2.89 ± 0.42	0.81 (.444)
	Bachelor’s degree^b^	2.98 ± 0.44		3.11 ± 0.65		3.60 ± 0.49		3.27 ± 0.85		2.86±0.54	
	Master’s degree^c^	3.19 ± 0.27		3.69 ± 0.28		4.01 ± 0.51		3.69 ± 1.01		3.06±0.33	
Religion	Yes	2.97 ± 0.47	−0.32 (.748)	3.29 ± 0.72	2.37 (.018)	3.68 ± 0.48	0.95 (.341)	3.31 ± 0.89	0.45 (.652)	2.88±0.55	0.23 (.814)
	No	2.99 ± 0.41		3.07 ± 0.62		3.61 ± 0.50		3.26 ± 0.83		2.87±0.50	
Experience of a family member`s death	Yes	3.06 ± 0.37	1.19 (.235)	3.06 ± 0.64	−0.75 (.453)	3.69 ± 0.54	1.00 (.315)	3.23 ± 0.83	−0.38 (.699)	3.02 ± 0.40	2.10 (.036)
	No	2.97 ± 0.44		3.14 ± 0.66		3.61 ± 0.48		3.28 ± 0.85		2.84 ± 0.53	
Total clinical career (yr)	1~4^a^	2.98 ± 0.42	1.79 (.149)	3.14 ± 0.70	0.42 (.733)	3.65 ± 0.51	0.62 (.597)	3.14 ± 0.84	3.49 (.016) a < d	2.90 ± 0.52	2.37 (.070)
	5~9^b^	3.06 ± 0.44		3.13 ± 0.56		3.57 ± 0.50		3.31 ± 0.90		2.91 ± 0.46	
	10~14^c^	2.98 ± 0.45		3.02 ± 0.66		3.61 ± 0.45		3.43 ± 0.72		2.82 ± 0.54	
	≧ 15^d^	2.80 ± 0.35		3.20 ± 0.71		3.74 ± 0.51		3.76 ± 0.67		2.57 ± 0.57	
Experience of emergency department work (yr)	1~4	2.99 ± 0.42	0.69 (.553)	3.12 ± 0.68	0.12 (.944)	3.66 ± 0.50	1.00 (.393)	3.23 ± 0.85	0.93 (.423)	2.90 ± 0.51	0.87 (.455)
	5~9	3.01 ± 0.46		3.14 ± 0.59		3.54 ± 0.46		3.30 ± 0.88		2.87 ± 0.52	
	10~14	2.98 ± 0.39		3.15 ± 0.61		3.67 ± 0.57		3.38 ± 0.73		2.76 ± 0.50	
	≧ 15	2.73 ± 0.39		2.97 ± 0.85		3.61 ± 0.19		3.79 ± 0.29		2.65 ± 0.68	
Number of end-of-life care experiences within 3 months	1~4^a^	2.96 ± 0.42	2.62 (.051)	3.09 ± 0.66	1.37 (.250)	3.57 ± 0.48	2.09 (.101)	3.36 ± 0.80	3.03 (.030) a > d	2.78 ± 0.57	3.70 (.012) a < d
	5~9^b^	2.97 ± 0.36		3.08 ± 0.54		3.65 ± 0.48		3.26 ± 0.75		2.94 ± 0.35	
	10~14^c^	2.97 ± 0.45		3.13 ± 0.66		3.62 ± 0.44		3.31 ± 0.76		2.93 ± 0.45	
	≧15^d^	3.21 ± 0.49		3.37 ± 0.78		3.84 ± 0.63		2.82 ± 1.18		3.10 ± 0.50	
Efforts to provide explanations and education about end-of-life care	Very hardworking^a^	3.09 ± 0.52	0.67 (.569)	3.35 ± 0.57	4.57 (.004)a > c	3.98 ± 0.50	10.74 (< .001) a > c	3.06 ± 1.03	0.78 (.504)	3.17 ± 0.29	3.90 (.009) a > d
	Somewhat hardworking^b^	2.98 ± 0.42		3.16 ± 0.65		3.64 ± 0.47		3.28 ± 0.82		2.85 ± 0.51	
	Somewhat un-hardworking^c^	2.97 ± 0.40		2.77 ± 0.61		3.29 ± 0.42		3.40 ± 0.79		2.77 ± 0.60	
	Not hardworking at all^d^	2.80 ± 0.09		2.97 ± 0.27		3.47 ± 0.64		3.36 ± 0.87		2.62 ± 0.20	
Experience of education about end-of-life care	Yes	2.98 ± 0.46	−0.17 (.862)	3.10 ± 0.67	−0.38 (.699)	3.67 ± 0.49	1.04 (.296)	3.29 ± 0.89	0.26 (.791)	2.87 ± 0.55	0.06 (.946)
	No	2.99 ± 0.41		3.14 ± 0.64		3.60 ± 0.50		3.26 ± 0.82		2.87 ± 0.50	
Total score		2.99 ± 0.43		3.12 ± 0.65		3.63 ± 0.49		3.27 ± 0.84		2.87 ± 0.51	

M = Mean; SD = Standard deviation.

**Table 3.** The Relationship between Emergency Room Nurses' Perceptions of a Good Death, Sense of Calling, Empathy Competence, End-of-Life Care Stress, and Attitudes toward End-of-Life Care (N = 248)

Variables	1	2	3	4	5
	r (*p*)				
1. Perceptions of a good death	1	–	–	–	–
2. Sense of calling	.32 (< .001)	1	–	–	–
3. Empathy competence	.33 (< .001)	.32 (< .001)	1	–	–
4. End-of-life care stress	−.43 (< .001)	−.14 (.019)	−.19 (.003)	1	–
5. Attitudes toward end-of-life care	.48 (< .001)	.33 (< .001)	.24 (< .001)	−.44 (< .001)	1

**Table 4.** Factors Influencing Emergency Room Nurses' Attitudes toward End-of-Life Care (N = 248)

Variables	B	*β*	t	*p*
General characteristics				
Experience of a family member's death (Yes = 1)	0.15	.11	2.16	.031
Efforts to provide explanations and education about end-of-life care	0.08	.08	1.55	.122
Number of end-of-life care experiences within 3 months	0.04	.09	1.88	.061
Perceptions of a good death	0.33	.27	4.44	< .001
Sense of calling	0.14	.18	3.27	< .001
Empathy competence	−0.01	−.01	−0.08	.934
End-of-life care stress	−0.16	−.27	−4.75	< .001
R^2^ = .37, Adjusted R^2^ = .35, F = 19.72, *p* < .001				

## Data Availability

The data that support the findings of this study are available from the corresponding author upon reasonable request.
